# Home advantage mediated (HAM) by referee bias and team performance during covid

**DOI:** 10.1038/s41598-021-00784-8

**Published:** 2021-11-03

**Authors:** Merim Bilalić, Bartosz Gula, Nemanja Vaci

**Affiliations:** 1 Department of Psychology, University of Northumbria at Newcastle, Ellison Square, Newcastle, NE1 8ST UK; 2 Department of Psychology, University of Klagenfurt, Klagenfurt, Austria; 3 Department of Psychology, University of Sheffield, Sheffield, UK

**Keywords:** Psychology, Human behaviour

## Abstract

The fans’ importance in sports is acknowledged by the term ‘the 12th man’, a figurative extra player for the home team. Sport teams are indeed more successful when they play in front of their fans than when they play away. The supposed mechanism behind this phenomenon, termed Home Advantage (HA), is that fans’ support spurs home players to better performance and biases referees, which in turn determines the outcome. The inference about the importance of fans’ support is, however, indirect as there is normally a 12th man of this kind, even if it is an opponent’s. The current pandemic, which forced sporting activities to take place behind closed doors, provides the necessary control condition. Here we employ a novel conceptual HA model on a sample of over 4000 soccer matches from 12 European leagues, some played in front of spectators and some in empty stadia, to demonstrate that fans are indeed responsible for the HA. However, the absence of fans reduces the HA by a third, as the home team’s performance suffers and the officials’ bias disappears. The current pandemic reveals that the figurative 12th man is no mere fan hyperbole, but is in fact the most important player in the home team.

## Introduction

In soccer, and other sports that feature eleven field players per team, the fans are often called “the 12th man”, explicitly acknowledging the importance of fans' support in a team’s success. Indeed, research shows that regardless of the sport and period of time, teams are more successful when they play at home than when they play away^1–3^. The most important factor behind this phenomenon, called the home advantage (HA), is believed to be the presence of the figurative 12th man^4^. The behavior of the supporting home crowd spurs the home team to perform better and possibly even bias referees’ decisions towards their team, which in turn contributes to the favorable outcome for the home team. The exact influence of the spectators on the HA is difficult to disentangle because teams normally play in front of spectators, whether it be their own fans at home games or opposing fans at away games. The necessary control condition, playing at home and away with no crowd, has normally not been available. The current Covid-19 pandemic provides a unique opportunity for a naturalistic experiment given that some sport competitions continued behind closed doors. Here we analyze over four thousand soccer games from twelve European leagues and present a new model—the Home Advantage Mediated model (HAM)—showing that the fans are indeed responsible in large part for the HA, albeit indirectly, through more dominant in-game performance of the home team and bias from the officials. The overall magnitude of the home venue advantage was reduced by almost a third when the games were played without fans.

### Theoretical explanations of HA

The classical framework^4^ for explaining HA assumes that venue factors influence physiological and psychological states, which in turn determine the outcome of the game. The most obvious and important venue factor is the crowd, but travel and familiarity also play a role. For example, the away teams need to travel to the venues, which will inevitably mean that they will be more tired than the home teams. Studies have shown that the travel factor is an important predictor of the HA, but only when the teams need to travel through two or more time zones^5–7^. Similarly, the home team’s familiarity with the venue, such as the dimensions of the pitch, should be beneficial to their performance. Research into sports such as baseball, where facilities vary greatly from one venue to another, did not show that the HA is more pronounced than in sports where the rules (e.g. dimensions of the courts) are uniform, such as basketball^8^. However, teams do perform significantly worse in the season when they move to a new stadium compared to their performance at the old stadium^9,10^.

Familiarity and travel influence the HA, but their impact is arguably overshadowed by the influence of home fans. The greater the number of fans in the crowd, the bigger the HA^8,11–14^, particularly when the crowd was dense^8,15^ and close to the playing field^16^. The noise generated by the home fans, and other demonstrations of support, alter physiological processes in both home and away team members^17^, which then influence their psychological states. For example, club-level soccer players were found to have higher levels of testosterone a couple of hours before kickoff in home games compared to their levels before training sessions or even away games^18^. Similar differences were found in junior hockey players, but there was a decrease in testosterone levels before away games rather than an increase before home games^19^. Testosterone is also higher after a win at the home venue than after a win at an away venue^20^.

Increased levels of testosterone are generally related to aggressive behavior^21^. Given that a higher level of testosterone is associated with higher metabolic rate of muscles, in the context of sport this may mean that home players are better equipped for necessary physical aggression and generally more motivated to compete^18,22^. This line of evidence is consistent with an evolutionary-based territoriality model where these responses are seen as natural responses to protecting one’s territory^18,23^. There is indeed evidence that observers rate the same players as more aggressive, assertive, and dominant when playing at home than when they compete away^24^. Self-reports also indicate that confidence is higher before home games whereas anxiety is lower^25,26^. These physiological and psychological factors are then responsible for how much team members exert effort, that is, how much they run, how aggressive and dominant they are, and possibly how well they deal with adversity. These team performance indicators are directly related to the outcome and therefore to the HA.

The crowd is also believed to influence the behavior of coaches and referees. When playing at home, coaches set more challenging goals and choose more offensive playing tactics, possibly because they have higher expectations of winning^27^. The few experimental studies on referees’ decisions found that the crowd noise does indeed favor the home team: fewer punishments are administered to the home team in comparison with situations where the crowd noise was absent^16,28^. Although not a formal part of the traditional model, it is known that performance itself influences referees’ decisions^29–31^. Attacking teams may force, for example, the other team to revert to fouling, which results in more warnings for the defending team^32^.

### Problems with previous accounts of HA

**Figure 1:**
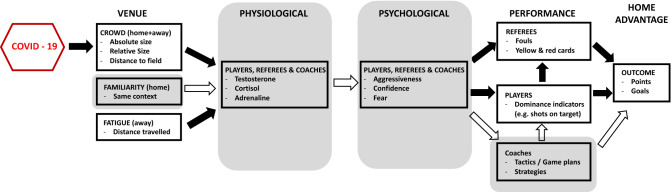
Home Advantage Mediated (HAM) model. The crowd presence influences how players, referees, and coaches feel (physiological and psychological factors), which in turn influences their performances, which are then acting as the determinants of the outcome. In the traditional framework^4^, the performances of players, referees, and coaches are incorporated in the outcome/HA. Here they are an independent factor, which mediates the influence of physiological and psychological factors (and consequently the influence of the crowd). The factors with the grey background (e.g., physiological, psychological, and coaches’ choices), and their relations indicated with empty white arrows, were not assessed directly in this study. For the sake of conciseness, we present the models including the fatigue (distance) factor in the Supporting Information (SI), Section 10.

There are several problems with the current accounts of HA. First, the team performance indicators (e.g. number of attacks, shots—in one word, dominance) are often taken as proxies for the outcome. They are, however, a crucial independent factor that needs to be separated from the actual outcome. For example, less dominant defending teams, which are generally away teams, tend to receive more warnings as they try to fend off the more dominant attacking home teams^29,30^. In other words, team performance not only influences the outcome directly, but it also affects other factors, such as referees’ decisions. A complete model needs to account for this and other interdependencies between factors (see Fig. 1).

Second, most relevant factors have been investigated separately^11,28,32–36^. This is unfortunate as it could lead to wrong conclusions. For example, an apparent referees’ bias could simply be a natural consequence of the in-game performance of teams—less dominant, defensive teams forcing referees’ decisions. Other in-game contextual factors may also influence the dynamics of referees’ and teams’ behavior as the game unfolds. It has been suggested, for example, that referees may switch between rule-based decision-making and mere game management depending on the context such as score and/or crowd behavior^31,37^. In rare cases where they have been examined concurrently, the assumed mechanism (e.g. indirect influence of the crowd on results through team and referees’ performance) has not been specified^13,29,38–41^. We do not know whether the HA is a consequence of referees’ behavior independent of the team performance, nor do we know to what extent referees’ behavior and/or team performance cause HA. This trend has continued in the latest studies, which also analyzed the last season affected by Covid-19^39,41–52^. While most of these studies demonstrate a reduction in HA due to the absence of a live audience, the processes behind the reduction are unknown. Whether and to what extent referees’ bias and team performance cause the HA reduction is impossible to answer.

Finally, previous research lacked a control condition to complete a proper falsification design^53–55^. A control condition is an important and necessary step to demonstrate better performance at home than away if we want to conclude that the crowd causes the HA. In order to infer causation, however, one also needs to demonstrate that the absence of home fans diminishes the phenomenon. Due to natural constraints, this has rarely been possible, as the crowd is generally present in both home and away games. In rare instances where this was not the case, the sample size of games without the fans was rather small^56–58^.

### Home advantage mediated (HAM) model

Here we propose a new theoretical model to simultaneously investigate the direct and indirect effects of the home venue and crowd on the HA (see Fig. 1). We call this new framework the Home Advantage Mediations (HAM) model, because it is based on the theoretically assumed indirect relations, that is mediations, between the concepts. For example, the crowd determines the outcome indirectly by biasing referees’ decisions (through changing their physiological and psychological states), which then influence the outcome of the game. Similarly, the fans inspire home team players to a better (team) performance (again, altering their physiological and psychological states), which is then reflected in the final score. Referees’ behavior is assumed to be partially influenced by players’ performance, therefore enabling us to differentiate between the direct effect of fans on referees’ behavior and indirect through team performance (see Fig. 1).

We also provide a blueprint for how HAM model can be formally tested with conditional process analysis^59–61^, an analysis which simultaneously includes moderations and mediations, where all relations and proposed mechanisms are investigated simultaneously. It therefore disentangles the mechanisms behind the HA and estimates their relative importance. The HAM model combined with conditional process analysis will be able to differentiate between referee bias due to the presence of fans and referee bias induced by team performance. More importantly, it will pinpoint which of the factors, and to what extent, produce the HA in the first place. We will be able to say whether the processes that lead to team performance and/or referee bias are the real causes behind the HA. In that sense, the HAM follows theoretical and statistical models in sport psychology, which combine influences from social and cognitive psychology to specify dynamic (in-game) processes^31,37,62^.

### Current study

The model can easily accommodate new factors, such as the current pandemic which has forced sport competition to take place without fans and, therefore, created a natural experiment^63^. Only the inclusion of pandemic in the model, that is the absence of fans, enables us to provide causal evidence for the major role of the home fans in the HA. HAM includes the Covid factor, that is the presence and absence of fans, as a moderating factor. It influences directly only the Venue factor, but its indirect influence is then spread throughout all individual relations related to the Venue factor (see Fig. 1). For example, the home venue may induce more referee bias towards the away team and a better performance by the home team. The inclusion of the Covid factor in this relation would demonstrate whether those relations have been altered, that is moderated, by the absence of fans. Most importantly, the HAM model enables us not only to specify whether the absence of fans modified the HA, but also whether Team Performance and/or Referee Decisions are responsible for the reduction, and if so, to what extent.

Here we test out the HAM model by analyzing the 2019/2020 season in European soccer, which took place with and without spectators due to Covid-19. The results of teams in the early part of the 2019/2020 season played in front of spectators serve as a control for the results of the same teams in the latter part of the 2019/2020 season when no spectators were allowed. If the crowd is one of the driving factors of the HA, the overall advantage of the home team should significantly decrease in the post-Covid period without the presence of the fans compared to the normal pre-Covid circumstances in which spectators were present. More specifically, the absence of the spectators should lead to worse in-game team performance by the home team as well as less bias in referees’ decisions, which in turn should weaken the overall influence of the home venue on the outcome.

## Results

### Descriptive analysis

**Figure 2:**
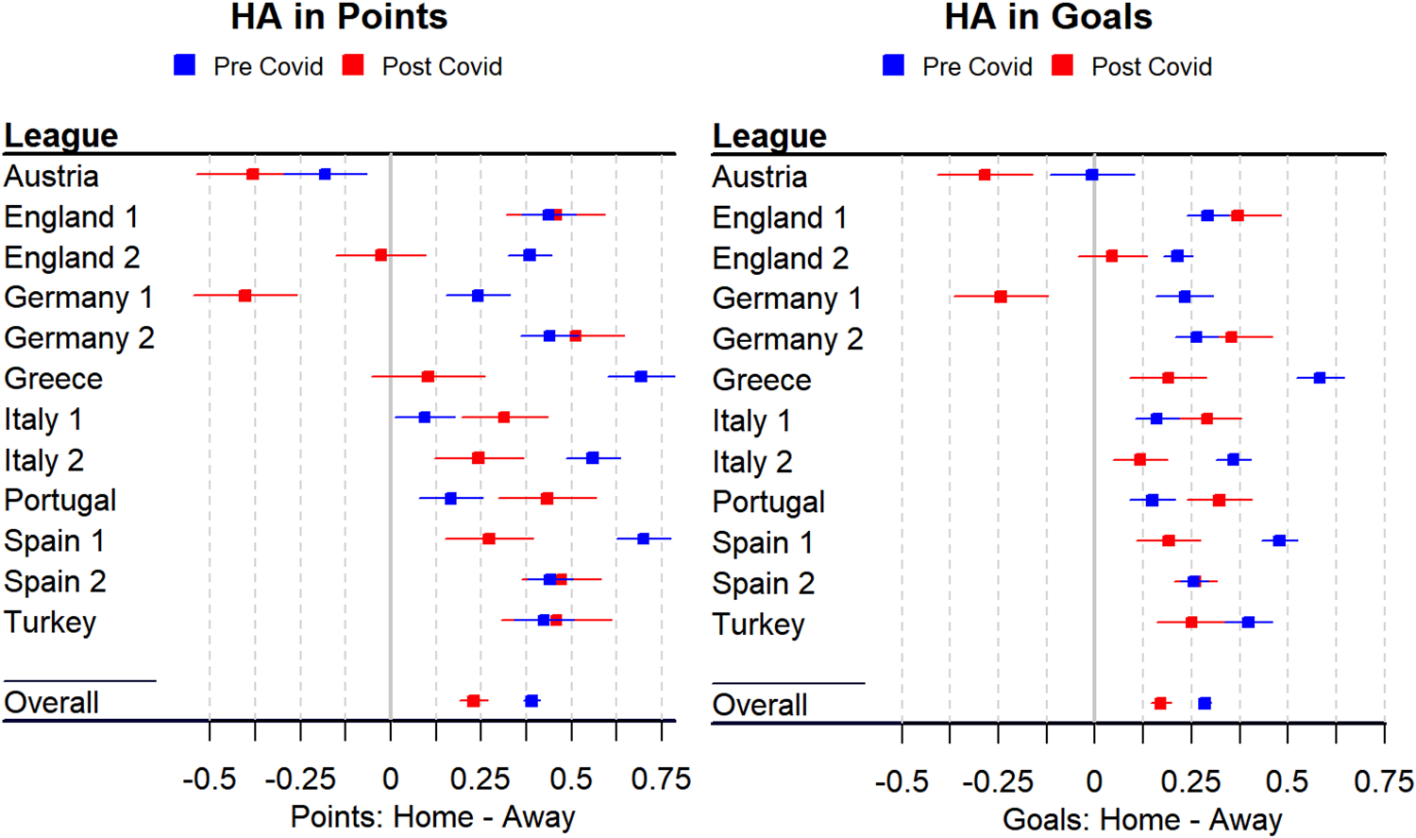
HA for different European leagues pre- and post-Covid. HA in Points (left panel): the difference in points won between home and away team for pre- and post-Covid periods; HA in Points (right panel): the difference in goals scored between home and away team for pre- and post-Covid periods. Error bars represent + /– 1 standard error of the mean. Number next to the country name (e.g., England 1) denotes competition tier (e.g. Premier League).

In the 2019/20 soccer season, 4356 matches were played by 224 individual teams in 12 different leagues in 8 different European countries (according to the professional football reference website—https://www.fbref.com). More than a quarter of these, 1131 matches, were played behind closed doors with no spectators present, which allows us to compare the performance of the home and away teams with and without the presence of the fans within a single season. Most of the 12 European leagues included in the analysis displayed reduced HA as measured by points won (Fig. 2, left panel) and goals scored (Fig. 2, right panel) in the post-Covid period compared to the pre-Covid period (see also SI, Section 4 for a robustness check). The biggest reductions in the HA due to the absence of the spectators were seen in England’s Championship (second tier), Germany’s Bundesliga (I division) and Spain’s La Liga (I division). The HA was somewhat increased in Portugal’s Premeira Liga and Italy’s Seria A (I division). While it is difficult to find any geographical trends, our robustness check of leaving one league out, confirms that the pattern of results was not driven by a specific outlier league (see Supporting Information, SI, Section 4.4).

**Figure 3:**
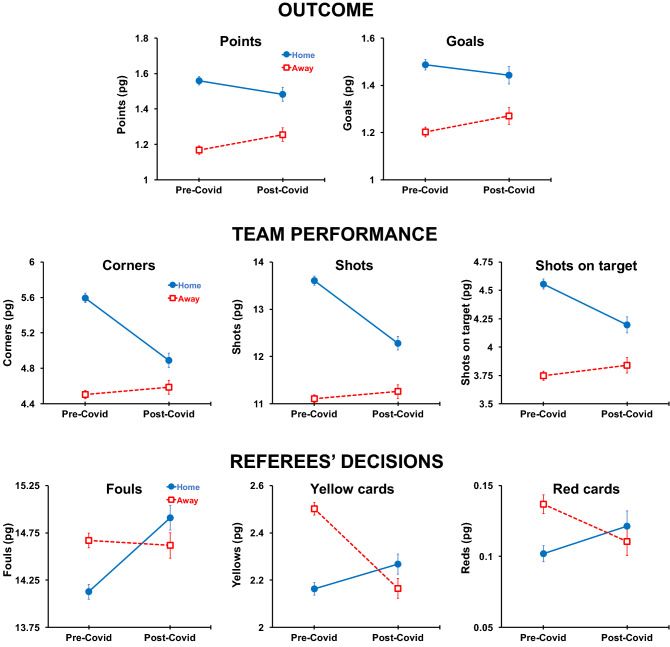
Descriptive Statistics for Venue x Covid. Outcome (upper panel): average number of points won per game (left) and average number of goals scored per game (right); Team Performance (middle panel): corners per game (left), shots per game (middle), and shots on target (aimed at the goal area) per game (right); Referees’ Decisions (lower panel): fouls per game (left), yellow cards per game (middle), and red cards per game (right). pg = per game (average values). Error bars represent + /– 1 standard error of the mean.

Figure 3 (upper panel) confirms that overall there was a big reduction in the HA in the games played without spectators. Home teams gained more points than away teams when they were supported by the fans, 0.39, than when they had to play without fan support, 0.23—a decrease of 41%. Home teams also scored on average 0.29 goals more than away teams in the pre-Covid period, whereas the same difference was only 0.17 goals in the games played without spectators (a drop of 41%).

These results are in line with other recent studies on the HA during the Covid period who did not use necessarily same samples^39,41,48,49,51^. For example, the drop from pre- to post-Covid in points was between − 0.15^41^ and − 0.24^48,51^, which includes our − 0.16 estimate (see Fig. 3). The home teams won on average between 2.6^39^ and 3.8%^48^ fewer games when they played without fans, which is in line with our estimates (2.2%). The drop in the home-away team difference in the goals scored between pre- and post-Covid periods in other studies is between − 0.08^39^, − 0.11^41^, and − 0.15^51^ goals, whereas out estimates of − 0.12 is between these estimates (see Fig. 3; the estimate which includes the leagues with partial attendance is − 0.10—see SI, Section 4.2).

One of the reasons for the reduced HA in the post-Covid period could be a weaker performance by the home teams in the absence of their fan support. Match statistics, in particular those related to created opportunities to score, such as corners, shots, and shots on target, are good indicators of team performance in soccer^13,35^. Teams that produce more opportunities to score are generally superior and more dominant teams. Figure 3 (middle panel) shows that there is a marked drop in home teams’ performance in three such team performance indicators. Home teams won fewer corners (− 0.70) when they played without the fans than when they played with fans, created fewer shots (− 1.32), and fewer of these shots landed on target (− 0.35). In contrast, away teams’ performance only slightly improved when the games were played behind closed doors (+ 0.09, + 0.15, and + 0.09 for corners, shots, and shots on target, respectively).

These statistics are in line with other studies which found that the advantage of the home team over the away team in corners taken diminishes for about 0.80^51^—it was 0.70 in our study (see Fig. 3). The home team advantage for shots also diminishes for about 1.35^41,51^ shots per game from pre- to post-Covid period, not unlike 1.47 in our study. The reduction in shots on target in our study (0.44) is also similar to that in other recent studies (0.35^51^ and 0.41^41^).

The other factor of the HA which is assumed to be affected by the spectators is the referee’s decision-making regarding official warnings: fouls (for rule infringements), official warnings (yellow cards), and immediate dismissals from the game (red cards). Figure 3 (lower panel) demonstrates that there were indeed considerable differences in the referees’ decisions in the pre- and post-Covid periods. More fouls by home teams were penalized by referees when they played without spectators (+ 0.78) than when they played with the fans present, whereas the away teams were whistled for a similar number of infringements (− 0.05). Home teams were punished more with yellow cards in the post- than in the pre-Covid period (+ 0.11), whereas the away teams received fewer official warnings (− 0.34) when the games were played without fans. Finally, the use of the most drastic punishment, the red card, was also influenced by the presence of fans—home teams received on average more red cards in the post-Covid period (+ 0.02) while away teams were punished less often (− 0.03).

Again, these statistics are in line with those found in other studies on the Covid. The favoring of the home team in terms of assessed fouls was reduced for 0.66^48^, 0.74^41^, and 0.88^51^ foul per game between the pre- and post-Covid periods—our estimate was 0.84 (see Fig. 3). A similar reversal in the yellow cards was established in other studies (0.36^39^, 0.40^48^, 0.48^41^, and 0.50^51^) as it is in our study (0.44). As in our study (0.04), the red cards were also assessed less often to the away teams in other studies—the reduction between 0.03^39,48,51^ and 0.05^41^.

### The home advantage mediated (HAM) model

The descriptive analysis demonstrated that the lack of spectators reduced the advantage of home teams across most European leagues in both the outcome and team performance. Home teams were also punished more often by the referees when they played without the support of their fans. We now investigate the interplay between these concepts in our HAM model using conditional process model analysis. We first created latent variables out of the three key factors in the theoretical model using factor analysis (FA) procedures (see Model Specifications in the Method). The Outcome was composed of goals and points, Team Performance was a latent factor of corners, shots, and shots on target, and Referees’ Decisions comprised fouls, yellow and red cards. Following the theoretical model (Fig. 1), we assumed that the Venue (home vs. away) influences the Outcome directly, but also indirectly through Team Performance and Referees’ Decisions. We also assume that Team Performance influences Referees’ Decisions and through them indirectly impacts the Outcome. The smallest units presented here are individual teams and their aggregated results (points and goals) when they played at home and away during the pre- and post-Covid periods (for a different approach which uses individual games as the smallest units of the analysis, see SI, Section 4.9).

**Figure 4:**
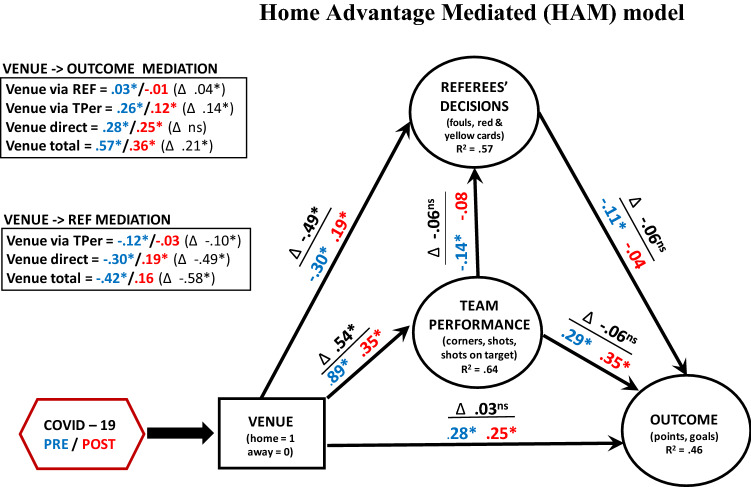
HAM Model—Bayesian (mixed-effects) conditional process analysis of HA. The interplay between Venue, Team Performance, Referees’ Decisions, and Outcome (the circular shape denotes latent variables—with the individual variables listed within the boxes). Lines with single-end arrows indicate the direction of influence. The numbers on the line are path model coefficients. The pre-Covid path coefficients are in blue, the post-Covid coefficients are in red, while their differences, indicated also by Δ, are in black. The statistically significant coefficients (95% credible intervals do not encompass 0) are indicated with *. The difference between the pre- and post-Covid path coefficients, delta (∆), is indicated above the individual coefficients (* when 95% credible intervals do not encompass 0 and ns when they do). The indirect influence of Venue on Outcome through Team Performance and Referees’ Decisions is formally tested in a mediation model (upper left box). The indirect influence of Venue on Referees’ Decision through Team Performance is also formally tested by mediation (lower left box). R^2^ is Bayesian full model coefficient of determination, which includes both fixed (COVID, Venue, Referees’ Decisions, Team Performance, Importance, and Rating) and random (league and team) effects. The control variables, Rating and Importance, were not presented here for the sake of conciseness (see SI, Section 4.1).

The model results confirm the descriptive analysis that playing at home leads to better Team Performance and fewer warnings from the officials (Fig. 4, path coefficients in blue for pre-Covid and in red for post-Covid). In return, better Team Performance leads to better results regardless of the period, while fewer official warnings in the post-Covid period lead to a more favorable outcome (a similar negative association was found in the pre-Covid period, but was not statistically significant). In other words, the modified classical theoretical model provides a good summary of the results (see R^2^ for Outcome, Referees’ Decisions, and Team Performance in Fig. 1; see SI, Section 4.1 for detailed assessment of goodness of fit).

Most importantly, the absence of the fans in the post-Covid period changed the dynamics in the model. Both Team Performance and Referees’ Decisions are significantly affected in the post-Covid period. The home teams are suddenly only half as dominant as with spectators (Venue → Team Performance + 0.89 vs. + 0.35 in the pre- and post-Covid periods, respectively—see also the difference indicated in the coefficient above in black). The same home teams that were getting fewer official warnings in the pre-Covid period are suddenly warned by the referees more often than the away teams when there were no fans present (Venue → Referees’ Decisions: − 0.30 vs., + 0.19 in the pre- and post-Covid periods, respectively). The influence of the team performance on the referees was also weakened in the post-Covid period but this difference was not significant (Team Performance → Referees’ Decisions − 0.14 vs., − 0.08 in the pre- and post-Covid periods, respectively).

These differences also resulted in the decreased overall influence of playing at home on the final result. While the indirect influence (i.e., mediation) of Venue on Outcome through Team Performance was significant for both the pre- and post-Covid periods, it was significantly larger when the teams were playing with the fans present (0.26 vs. 0.12—see the upper left box in Fig. 4). Similarly, the indirect effect of Venue on Outcome through Referees’ Decision was significant when the games were played with the fans present, but not when the games were played behind closed doors (0.03 vs. − 0.01), resulting in a significant difference between the two indirect effects. The overall influence of playing at home, which included both mediations through Team Performance and Referees’ Decisions, as well as the direct influence in the Outcome, was also (significantly) smaller by almost a third when the fans were absent (+ 0.36) than when they were present (+ 0.57).

The other factor that displayed a considerably different pattern of results due to the absence of fans was the Referees’ Decisions (see lower left box in Fig. 4). Not only was there a clear reversal of the direct trends in official warnings between home and away teams depending on the Covid period, but home teams were also considerably less dominant in the post-Covid period (0.89 vs. 0.35), which indirectly weakened their influence on the referees’ decisions. The mediation between Venue and Referees’ Decision through Team Performance was only significant when the fans were present (− 0.12 vs. − 0.03).This all resulted in a total effect of Venue on Referees’ Decision being not only almost three times greater when the fans were present than when the games were played behind closed doors, but also having the opposite influence—home teams, which usually enjoyed fewer official warnings when the fans were present, were suddenly punished more when there was no crowd (− 0.42 vs. + 0.16).

Both Referees’ Decisions and Team Performance had associations of a similar magnitude with the Outcome in the pre- or post-Covid period, whose difference was consequently not significant. This pattern of results is expected. Team Performance and Referees’ Decisions factors may be altered by the absence of spectators. However, their direct influence on the Outcome should be similar irrespective of the Covid period. If a team performs better and receives fewer warnings, the outcome will be similarly positive regardless of whether or not they played in front of fans.

### Additional analyses and controls

We used two control variables in all our analyses: (team) Rating, which indicates how strong an individual team is before the match, and (match) Importance, which indicates how important that match is for the team (e.g., whether it is a championship match, a crucial match in a battle to avoid relegation from the league, or a match with no particular significance). These control variables played important roles in predicting the Team Performance, Referees’ Decision, and Outcome (see SI, Section, 4.1). However, there is no interaction between either Rating or Importance with the Covid variable (see also SI, Section 4.1). In other words, the HA reduction in the post-Covid period is not a consequence of differing schedules in the pre- and post-Covid periods (see SI, Section 5).

In the main analysis we used only the leagues which excluded all spectators in the post-Covid period. The inclusion of the leagues which partially allowed fans during the post-Covid period produced similar results, but the overall effects were somewhat diminished (SI, Section 4.2). Our own analysis of the recently finished European Championship, which also allowed partial attendance and featured home matches, indicates that even partial attendance contributed to the advantage of home teams (SI, Section 4.3).

Other additional analyses feature robustness and sensitivity checks such as: leaving out individual leagues from the analysis (SI, Section 4.4), use of individual games instead of individual teams (SI, Section 4.9), points and (expected) goals as the outcome measures (instead of their latent construct—SI, Section 4.8); home-away difference as the measures of the outcome (SI, Section 8); controls for the use of the Video Assistant Referee (VAR) in the leagues (SI, Section 4.5) and for individual differences among referees (SI, Section 4.10); the addition other factors such as distance, attendance, and stadium characteristics (SI, Section 10); the specification of alternative paths in the model (SI, Section 4.7); the investigation of dynamic within game factors with additional team performance indicators (SI, Section 9); and the use of non-Bayesian approaches (SI, Section 6) and different statistical techniques (SI, Section 7). All our additional analyses, some with vastly different analytical approaches, others with differing statistical philosophies, corroborate the main conclusions from the results presented here.

## Discussion

The current pandemic provided a unique opportunity to systematically investigate whether the crowd presence drives the well-known and robust phenomenon of the HA. We demonstrated that this is indeed the case in European soccer as the advantage of the home teams was reduced by one third when the figurative 12th man was missing.

### HA mediated by team performance and referees’ decisions

According to our model, the HA reduction is entirely the consequence of the crowd’s indirect influence on the result through the home team’s performance and referees’ decisions. The fans spur the home team towards better performance, presumably by altering home team players’ physiological and psychological states^23^. These physiological and psychological factors are responsible for how much team members exert effort, that is, how much they run, how aggressive and dominant they are, and possibly how well they deal with adversity^22^. Once the fans were not present, the effect on the physio-psychological factors is absent and the team performance is consequently significantly weakened. Our results indicate that the extent of the home venue on the home team’s dominance (Venue → Team Performance) is more than halved. Most importantly, the absence of spectators impacts only the home team, which performs considerably worse in the post-Covid period, unlike the away team whose performance remains the same or slightly improves (see Fig. 3). This is in line with previous research showing the altered physio-psychological states of the players when they play at home but not when they play away^18,26^. More broadly, it is also consistent with an evolutionary-based territoriality model where these responses are seen as natural responses to protecting one’s territory^23^.

Similarly, the fans bias referees’ decisions in favor of the home team (Venue → Referees’ Decisions), possibly through social pressure where the generated noise serves as the cue for decision^16,17,31^. Once the noisy crowd is not present, referees’ decisions are not only more balanced, but even favor the away team. Although the referees’ decisions may not have as large an effect on the outcome as the team performance, it is still a substantial effect as Referees’ Decisions also contributes almost 20% to the overall reduction in the HA (Δ 0.04 / Δ 0.21—see the upper box in Fig. 4). Most remarkably, the referees’ effect is still present to such a large extent even after we have accounted for the team performance, which is arguably the most important predictor of the outcome (and closely related to Referees’ Decisions). Consequently, the calls^4^ for removing the referee’s decisions from the theoretical model may be premature.

### Alternative interpretations

The magnitude of the reduction due to the absence of fans is, however, surprisingly large in both Team Performance and Referees’ Decisions. Team Performance of the home team was almost three times worse when they played behind closed doors than when they played in front of their fans (see Fig. 4, Venue → Team Performance relation). A similar reduction was also found in the Referees’ Decisions (see Fig. 4, lower left box), where in fact a reversal of fortunes was present—the same home teams which were enjoying milder treatment from the officials were suddenly receiving significantly more warnings in the post-Covid period. It is possible that these drastic changes are interconnected. The reversal in the official warnings may not be a consequence of diminished team performance in the post-Covid period, as this factor has been included in the model and therefore partialed out from the Venue → Referees’ Decisions relation (see Fig. 3). However, it is possible that home teams may not be able to be as aggressive as they are used to being when the fans are present^23,24,27^, as the referees are stricter in sanctioning the aggressive play of the home team when there are no fans. In this way, we may have a situation where home teams, which try to play as aggressively as they do in front of their fans, are punished by officials, which in turn inhibits their team performance.

The analysis with the alternative causal flow, from Referees’ Decision to Team Performance, does not change the main conclusion (SI, Section 4.7). The Referees’ Decisions still play an important role in the overall HA reduction due to the lack of spectators. The other alternative model, which features possession as the predecessor for both Referees’ Decisions and the team performance indicators (corners and shots), may be a more realistic depiction of the causal flow (SI, Section 9). The model with possession and additional indicators of team performance is, however, somewhat domain-specific, and it does not improve the statistical fit of the model despite the added complexity. The direction of the relation between team performance and referees’ bias may be too difficult to disentangle at the macro level of a season. Even at the level of a single game, it may be challenging to find the correct causal flow as there is no detailed information on the time course of the relations^31,37,64,65^. Future studies could investigate details of individual games to gain insight into this intriguing possibility.

Nevertheless, playing at home had a direct effect on the outcome even when the indirect effects through team performance and referees’ decisions were accounted for (Venue → Outcome relation in Fig. 4). Given that the magnitude of the direct effect was unchanged when spectators were absent, it is tempting to associate this influence with other venue-related factors such as familiarity^9,10^. Familiarity is, however, theoretically associated with team performance and not supposed to directly influence the outcome. Similarly, the factors of travel and attendance were also most likely not responsible for the direct effect of the home venue on outcome as they did not have either direct or indirect effects (via performance and referees’ decisions) on the results (see SI, Section 10). The direct effect of venue on outcome is more likely to be the variance which our model could not capture through intermediate variables such as team performance and referees’ decisions. The SI (Section 9) demonstrates that when additional team performance indicators were added to the model, not only was the model not fitting better or explaining more variance, but the direct effect was still present. It is possible, however, that other fine-grained indicators, such as, for example, location of shots taken, may improve the predictive power of performance indicators and consequently decrease the unexplained direct effect of the home venue.

### Shortcomings

As with any natural experiment, the resumption of sporting activity without fans during the pandemic also had shortcomings. Arguably the biggest of these is that the teams played different opponents in the pre- and post-Covid periods. Our analysis, however, rules out the differing schedules in the pre- and post-Covid periods as an explanation for the reduced HA (SI, Section 5). Similarly, we know that almost 50% of goals scored are not planned but a product of situational luck, something that also varies within a single season^66^. Our additional analysis on expected goals (SI, Section 4.8), which are calculated using objective shots’ characteristics, at least alleviates these concerns to some extent (assuming the emergence of these shots is not completely random).

The games were often played more frequently and allowed for more substitutes during the post-Covid period. Anxiety about the potentially deadly virus may also have affected the performance of players. These factors affect both home and away teams, but it is possible that the performance of a home team, which under normal circumstances is the more aggressive of the two, is more affected^23^. This speculative assumption is consistent with our results where only the home team is experiencing a decrease in performance during the post-Covid period, whereas the performance of the away teams remains constant (Fig. 3). Future, preferably experimental, investigations in line with previous research on the physiological and psychological factors^18–21,24–26^ may provide more insight into this intriguing possibility.

### Comparison with other studies and partial attendance

Previous research^56–58^, which used only a handful of matches without spectators, has produced mixed results. There was no significant reduction in HA when the games were played behind closed doors, but the referees’ bias towards away teams was attenuated^58,67^. The recent studies^39,41–52^ feature much larger samples and converge on the finding that the absence of fans does indeed significantly reduce the HA. Our results are in line with these recent studies on the HA during the Covid period (see the review in the Results). The slight differences in estimates are probably related not only to the differences in samples, but also to how the pre-Covid estimates have been calculated. Wunderlich and colleagues^41^, as well as Bryson and colleagues^39^, demonstrated that the HA reduction is considerably larger when the previous seasons are included than when only the 2019/20 season is used for the analysis. This is in line with the observed phenomenon of decreasing HA over time^68^ and was possibly a reason for the high estimates in some of the studies^48,51^.

A recent review^69^ found that majority of the Covid-related studies on HA found a statistically significant reduction in HA despite vast differences in sample, methodology, and statistical analysis. Even the studies which did not find the statistically significant HA reduction found that the HA was bigger in the pre- than in the post-Covid period. For example, Wunderlich and colleagues^41^ found in the 2019/20 season a reduction of 16–18% in the HA measured by goal and point differences between the home and away team (see SI, Section 8, for a similar model). Bryson and colleagues^39^ found in the same 2019/20 season a goal difference (0.08 per game) between home and away teams in pre- and post-Covid periods which just failed to reach the conventional significance level (it was significant at *p* < 0.10).

One possible reason for these results is that the pre-Covid period of the 2019/20 season produced markedly smaller HA than in the previous seasons (see Fig. 1 in Wunderlich and colleagues^41^). This is most likely a random fluctuation (see Fig. 2 in Wunderlich and colleagues^41^), but the already small starting value of the HA makes it more difficult to find a large suppression effects due to the lack of the spectators. The other possible reason is the samples these two studies used. Wunderlich and colleagues^41^ analyzed a sample that did not include some European leagues (e.g. Austria and Greece), which may have reduced the power to discover the significant HA reduction (see also Fig. 2 for HA in those two countries). Bryson and colleagues^39^ included these leagues in their analyses, but did also incorporate the leagues which allowed some fans to attend the games in their analysis. Their own additional analysis demonstrates that the leagues with partial attendance produce a somewhat different pattern of results from the leagues without fans (see their Table A7^39^). This was also confirmed in a recent study^51^ where the partial attendance and non-attendance was directly included in the analysis. The HA reduction was smaller in the leagues with partial attendance compared to the leagues with no attendance.

Our own analysis shows that the inclusion of the leagues with partial attendance during the Covid period somewhat weakens the HA reduction (see SI, Section 4.2). The recently finished European Championship, which also featured several matches in which some national teams played in their stadium with support from their fans, provides another piece of evidence that even partial attendance produces sizable HA effects. The national team playing at home scored on average almost half a goal (0.44) more per game than the visitors. The magnitude of the home advantage was virtually identical when the same national teams were qualifying for the European Champion and playing in front of full stadiums (see SI, Section 4.3). Other studies investigating the effects of hosting the World Cup found similarly large HA effects^70^. Future research should consider the seemingly pronounced difference between partial and non-attendance.

## Conclusions

While the recent studies on the effect of the fans’ absence on the HA all converge on the main conclusion, they are silent about the processes that lead to the HA reduction. The HAM model clearly identifies the referees’ behavior and in particular team performance as the channels of the reduction. Our HAM model can easily accommodate other factors under the influence of the crowd, such as coaches (see Fig. 1) who set more challenging goals and choose more offensive playing tactics when they play at home^27^. We also demonstrate how other relevant crowd factors, such as the absolute size of the crowd, its density^8^, and proximity to the playing field^16^ can be incorporated in the model (see SI, Section 10). The model can be generalized to other sport domains because it features concepts such as team performance and referees’ decisions which are ubiquitous for any sport. Similarly, it should be straightforward to incorporate the physiological and psychological factors as additional levels into the HAM model, since these factors would also act as mediators between the crowd and the final outcome in our HAM model.

Combining our novel modeling framework, which enables for simultaneous estimation of all relevant relations, with the naturalistic experiment allowed us to quantify the influence of spectators on the HA as well as to disentangle the individual mechanisms at work. Our results point out that the figurative 12th man is not only very real when it comes to the home team’s performance but is also arguably the single most important player. In the age of the increasing commercialization and internationalization of the top soccer clubs, it must be reassuring for the local fan base that the success of clubs still depends largely on the fans’ support in the stadium.

## Method

### Sample

We analyzed 4356 individual games played by 224 individual teams from 12 different leagues spanning 8 different countries (English Premier League & Championship, German Bundesliga 1 & 2, Spanish La Liga 1 & 2, Italian Serie A & B, Portuguese Primeira Liga, Greek Super League, Turkish Super Lig, and Austrian Bundesliga). In total, 3225 games (74%) were played with spectators present (pre-Covid) and 1131 (26%) in the post-Covid period without spectators.

Our general strategy for determining the sample was as following. The leagues for which the necessary indicators (e.g., shots, fouls) were not freely available were excluded as they would be excluded automatically in the analyses (listwise deletions of missing values). The leagues that (partially) allowed fans into stadiums during the Covid-19 period were also excluded from the main analysis. While the inclusion of these leagues with partial attendance produces broadly similar results, the HA reduction was nevertheless suppressed (see SI, Section 4.2). This is in line with other research which explicitly investigated the differences^39,51^. We also provide a preliminary analysis of the recently finished European Soccer Championship to show that even partial attendance benefits home teams to a similar extent as the full attendance (see SI, Section 4.3). Finally, we decided to analyze all leagues together instead of investigating them separately as has recently been done in some studies^52^. The number of observations in the Covid period was rather small for individual leagues. Such small samples give rise to unreliable estimates which may or may not reflect the true nature of the influence of Covid. It is statistically more prudent to include all leagues, not only because the estimates become much more reliable, but also because we are dealing with top European leagues which, despite their differences, share much more commonalities with each other than, say, non-European leagues^2,71^. Our statistical analyses also explicitly model and account for the differences between leagues (see below).

The data on the individual games were obtained from the professional football reference website (https://www.fbref.com). The data includes the scores, goals for each team, shots, shots on target, corners, fouls, yellow and red cards, statistics on possession and passes and several other indicators for each individual game. For the Greek Super League, the data was supplemented using the football data website (https://www.football-data.co.uk) because the statistics on the model variables (e.g., corners, shots, shots on target, fouls, yellow and red cards) were missing in the professional football reference data. The data from pro football reference and football data websites were supplemented using the FiveThirtyEight database, which also included team ratings and the importance of the match for both teams (for more information, see SI and https://fivethirtyeight.com/methodology/how-our-club-soccer-predictions-work/).

The travel distances between the home and away teams, which were taken as a measure of fatigue for the away team, were obtained by a R-package gmapsdistance^72^ using the Google Maps Platform (https://developers.google.com/maps/documentation). The stadium characteristics were obtained using the information available at the tranferkmark.de site (e.g. https://www.transfermarkt.de/fc-bayern-munchen/stadion/verein/27/saison_id/2019).

### Analysis

#### Descriptive statistics

We first operationalized the main concepts in the model. The Venue was a factor variable with two levels: home and away. The results indicators, closely related points and goals (for intercorrelations, see SI, Section 1), were the Outcome. The Referees’ Decisions were measured by the indicators completely depended on the officials: fouls, yellow and red cards. Finally, the Team Performance was measured by three closely related indicators of team dominance: corners, shots, and shots on target. While it is also possible to cluster additional Team Performance variables into the indicators of possession (e.g., number of touches) and defense (e.g., long balls), adding these concepts into the model did not improve the model fit or its predictive power—the main results also remained the same (see SI, Section 9). Consequently, we have chosen the simpler model featuring only one Team Performance indicator for the main presentation.

#### The home advantage mediated (HAM) model

For the model, we conducted factor analysis (FA) on each of the three constructs separately, which confirmed a single factor structure for all three constructs (explaining 82%, 66%, and 49% of the variance for Outcome, Team Performance, and Referees’ Decisions, respectively—SI, Section 3). We then used the factor loadings for individual variables in a regression model to produce latent standardized variables of Team Performance, Referees’ Decision, and Outcome. The approach of reducing multiple variables to their latent concepts was chosen on the one hand to improve the capturing of the measured concepts^73,74^, and on the other, to reduce the complexity of the statistical model^75^. The FAs were conducted for all available individual data, irrespective of the leagues, because we also analyzed all data together. Conducting the FAs separately for each league produced practically the same results as the latent variables based on all available data were highly correlated with the latent variables that accounted for individual league (correlations > 0.95).

For our HAM model we used a game-pairs framework where a single game with its indicators (e.g. goals, points, team or referees’ performance) was in the analysis for both home and away teams for similar approaches, see^34,76,77^. The first variation, presented here in the main text (Fig. 4), uses averages across individual teams for their performance at home and away during the pre- and post-Covid periods. The second variation employs the granular analysis of individual games where each game, instead of the average for a team, is in the model (see SI, Section 4.9). The game-paired framework enables us to check whether the differences, for example, in the team performance (e.g., shots) are the product of a reduced performance in home teams, or increased performance of away teams, or a combination of both. Other approaches, such as using difference between home and away teams^13,39,48^ as the input values (and, therefore, leaving out Venue as a factor) yield essentially the same pattern of results (see SI, Section 8).

The specified model was then analyzed using conditional mixed-effects process analysis^60^ in the Bayesian framework^78^. We used a conditional process analysis because we wanted to investigate the assumed direction of the influence. Figure 1 (and also Fig. 4) illustrates, for example, that the Venue was supposed to influence Team Performance and Referees’ Decisions, which in turn influenced the Outcome. The Referees’ Decisions were influenced by the Team Performance too, and there was a direct influence of Venue on Outcome. The conditional process analysis on a model set up this way allows for testing the indirect effect of Venue on Outcome through Team Performance and Referees’ Decisions. In other words, one of the main theoretical questions, namely whether Team Performance and Referees’ Decisions mediate the influence of Venue, can be answered with our model. Similarly, we could incorporate the influence of the absence of fans (Covid factor) in the path analysis by checking the interaction of the Covid factor with individual path relations—also known as moderated mediation^59^. For example, theoretically, the absence of spectators should only affect the Venue related paths (to Team Performance and to Referees’ Decisions), but for the sake of completeness we allowed Covid to interact with other paths in the model. The model also included estimates of team strength and game importance to control for possible differences in the pre- and post-Covid (see SI, Section 4.1).

To control for possible differences in the pre- and post-Covid schedule and importance of the games, we used FiveThirtyEight’s team strength (rating) and game importance measures for both home and away teams for every match (for more information about the SPI and importance measures, see SI, Section 5). Both rating and importance were included in the model as additional factors. Both were assumed to influence the Outcome directly, as well as indirectly through Team Performance and possibly Referee’s Decisions. As with the previous paths in the model, the path of the rating and importance were also allowed to be influenced by the Covid factor (interaction, or moderated mediation). These interactions with Covid allowed for checking whether teams played stronger/weaker teams in the post-Covid period or had more important games where they exerted more effort. None of the interaction with Covid, either with Rating or Importance, was significant (see SI, Section 4.1).

The lowest units were the individual teams (for the analysis featuring individual games, SI Section 4.9, the smallest units were the individual games). These 224 individual teams were nested within 12 leagues, and both team and country factors were used as the random factors in the mixed-effects analysis^79^. By including the team and/or league level random factor we control for additional variability in the outcome influenced by random variations in the team performance or general differences between the leagues^80^. Using the random structure of the model allowed us to partially pool our coefficient estimates. The estimates for the leagues and teams with fewer data points or with unexpectedly large or small effects (e.g., outliers) were pooled towards the estimates from the groups with larger number of observations and the overall mean of the distribution. This is in contrast to the alternative specifications of model structure, such as including teams and leagues as fixed effect in the model (i.e., predictors). In the case of fixed-structure model, our coefficients would be estimated separately for each team and league, resulting in the no-pooling estimation of the model and increasing the chance of overfitting of the leagues and teams with smaller number of data points, especially in the post-Covid period when we overall have fewer data points. We expand on these points in the supplement (see SI, Section 4.1.1) while more detailed statistics are available in Online SI.

The Bayesian framework was chosen for its flexibility which enabled us to conduct all analyses within a single framework, as well as its ability to provide rich information about the model and its parameters^78^.

#### Additional analyses

The analyses presented in the main text are only a subset of a larger set of analyses presented in the SI and online SI. The additional analyses feature robustness checks, as well as different statistical and analytical approaches. We first present checks for partial attendance in our sample by including the leagues which allowed fans into stadiums (SI, Section 4.2), then analyze the recently finished European Championship to confirm that even partial attendance produces substantial HA (SI, Section 4.3). We then demonstrate that the results were not driven by individual leagues/outliers (by re-estimating the main model repeatedly each time by dropping one league from the data set, SI, Section 4.4). The results also hold when we controlled for the use of Video Assistant Referee (VAR) in the leagues (SI, Section 4.5) or individual differences among referees (SI, Section 4.10), when we use the ratio of yellow cards and fouls as the referee indicators instead of the latent referee variable (SI, Section 4.6), and when we re-specify the causal path between referees and team performance (SI, Section 4.7). We then demonstrate that the results hold when we use the individual games (instead of the aggregated model across teams in Fig. 4), as well as when we use Points and Goals as separate outcome variables instead of the latent variable (see SI, Section 4). The results also hold when the common non-Bayesian approach is used, for example conditional path analysis (SI, Section 6). A different statistical technique, Structural Equation Modeling (SEM), where all the indicators of the concepts were incorporated directly in a single model, also produced essentially the same results (SI, Section 7). Similarly, using the differences between home and away teams^13,39,48^, instead of having them as aggregated pairs variables in the model, resulted in the same pattern of results (SI, Section 8). We also demonstrate that neither fatigue as measured by the distance traveled nor the relative number of fans present at the game had significant influence on the Team Performance, Referees’ Decision, or the Outcome itself (see SI, Section 10). Similarly, the stadium characteristics, such as the pitch size, pitch surface, and the presence or absence of a running track, did not significantly influence the Outcome. Only the absolute number of fans was significantly related to the Outcome both directly and through its positive relations with Team Performance. The effect is, however, most likely driven by team strength as better teams tend to have larger stadiums and consequently more fans (see SI, Section 10). Finally, adding additional Team Performance variables, such as possession or defense-related indicators, did not change the results (nor did they improve the model fit—SI, Section 9).

## Supplementary Information

Supplementary Information.

## Data Availability

The data and the code used for the analyses can be retrieved from https://osf.io/w52tc/?view_only=65abeb3c16504c789b54a3124f330e58.
