# Supplementary material for: Home advantage mediated (HAM) by referee bias and team performance during covid
Source: Sci Rep. 2021 Nov 3;11:21558. doi: 10.1038/s41598-021-00784-8 (PMC8566522; doi:10.1038/s41598-021-00784-8)
Supplement: Supplementary file 1 — Supplementary Information. [file 41598_2021_784_MOESM1_ESM.docx]

Supporting Information

**Home Advantage Mediated (HAM) by Referee Bias and Team Performance During Covid**

Merim Bilalić^1^*, Bartosz Gula^2^, Nemanja Vaci^3^

* Corresponding Author:

Merim Bilalić, Northumbria University, Department of Psychology, Ellison Square, NE1 8ST Newcastle, UK, +44 191 227 3291; **Email:**  [merim.bilalic@](mailto:xxxxx@xxxx.xxx)northumbria.ac.uk

**This PDF file includes:**

Supplementary text

Figures S1 to S14

Tables S1 to S19

SI References

**Other supplementary materials for this manuscript include the following:**

<https://osf.io/w52tc/?view_only=65abeb3c16504c789b54a3124f330e58>

Supplementary Text

1. **Descriptive Statistics and Intercorrelations**

Table S1 provides the mean and standard error of the mean (SE) for the Outcome (Points and Goals), Performance (Corners, Shots, and Shots on Target), and Referees’ Decision variables (Fouls, Yellows, and Reds) per game depending on the Venue (Home and Away) and Covid (Pre and Post).

**Table S1**. Descriptive statistics of Outcome, Performance, and Referees’ Decision depending on Venue and Covid. SE is standard error of the mean.

The other performance indicators, which we used for additional analysis, can be found in Table S2.

**Table S2**. Descriptive statistics for other performance indicators depending on Venue and Covid. SE is standard error of the mean.

Table S3 provides intercorrelations between all model-relevant variables presented in Table S1. Table S4 presents intercorrelations between all variables presented in Table S2, which feature additional team performance indicators.

**Table S3**. Intercorrelations between Output, Performance, and Referees’ Decision variables.

**Table S4**. Intercorrelations between additional team performance indicators.

1. **HA for Individual leagues**

Home teams were more successful than away teams in almost all individual leagues, with a slight exception of Austrian and Russian leagues (see Table S5). In most of the individual leagues (3/4), this home advantage decreased when the games were played without the audience.

**Table S5**. Home Advantage (HA) in individual leagues for pre- and post-Covid periods.

1. **Factor Analysis (FA) for latent variables**

**3.1. FA for Outcome, Team Performance, and Referees’ Decisions latent variables**

The outcome variable was composed of points won and goals scored. They were highly correlated (*r* = .65), and the latent factor based on these two variables explained 82% variance (see Table S6).

Similarly, the Referees Decisions were unambiguously represented by the variables of Fouls, Yellow and Red cards. These variables were also closely related, as reflected in their intercorrelations (*r* around .30) and the latent factor based on these three variables consequently explained 49% of the variance (Table S6). The penalty variable could also be considered a referees’ decision, but it correlated weakly with the other three variables (*r* < .05). It is arguably much more a product of the team performance (than fouls, yellow and red cards) as more dominant teams are more likely to create situations that would lead to penalties.

Finally, we decided on the variables Shots, Shots on Target, and Corners as the input variables of the latent factor of Team Performance. These three variables were highly related (*r* from .29 to .64), and their latent factor explained 66% of the variance (Table S6).

**Table S6**. Factor loadings and % explained variance for Outcome, Team Performance, and Referees’ Decisions latent variables.

The reasoning behind this decision was twofold. On the one hand, it is known^1^ that shots and shots on targets are good indicators of team dominance and, therefore the outcome (e.g. expected goals, xG, are calculated based on the shots and their location). On the other hand, we carried out exploratory factors analysis (FA) to determine the structure among the indicators. As Table S7 demonstrates, there were three latent factors: possession (total passes, pass successful, pass %, and touches), attacking dominance (shots, shots on target, and corners), and defense/set-pieces (crosses, areal duels won, throw-ins, and long balls). The corners variable was also together with the shot and shots on target variables (Factor 2). The cross variable was also related to the attack/dominance factor, but it was also related to the defense/set-pieces factor (Factor 3). In the end, we decided to represent the attack/dominance factor with the variables corners, shots, and shots on target as these variables were available for all individual leagues (e.g. the Greece first division did not have other team performance indicators).

The factors possession and defense/set-piece were also not included in the main model. This was done because the model with dominance/attack as Team Performance indicator was simpler and fitted the data better than the model with all three latent factors of team performance (see Section 9). We do, however, provide a possible theoretical model where possession influences dominance/attack and defense/set-pieces. As mentioned, this model doesn’t fit the data better than the simpler main model presented in the main text.

**Table S7**. Factor loadings for the individual team performance indicators

**3.2. Differentiation between Outcome and Team Performance Variables**

Outcome variables (Points and Goals) are sometimes considered as the same measure as Team Performance indicators (Corners, Shots, and Shots on Target). Theoretically, Team Performance indicators, however, precede the Outcome. In other words, the Outcome is a consequence of a teams’ performance. Here we show that the two concepts can also be statistically separated. Table S8 shows that Shots and Corners do not correlate highly with Points and Goals. The only Team Performance indication that correlates moderately is Shots on Targets.

We also used Confirmatory FA (CFA) to show that the two-factor model is better than the single-factor model (see Online SI). The single-factor model with all two Outcome and three Team Performance measures together did not have a particularly good fit (χ^2^(5) = 6002, p < .001, CFI = .66 and TLI = .32; RMSEA = .35, p < .05; SRMR = .14). The two-factor model, where Outcome and Team Performance were separately modelled, had a much better fit (χ^2^(4) = 2372, p < .001, CFI = .87 and TLI = .67; RMSEA = .25, p < .01; SRMR = .10). Most importantly, the formal difference between the fits of the two models was significantly different, indicating the two-factor model being the superior to the single-factor model (χ^2^(1) = 3630, p < .001).

**Table S8**. Intercorrelations and Factor loadings for the individual Outcome and Team Performance indicators

1. **Main model**

**4.1. Main model**

We used the brms R-package^2^ for running the Bayesian analysis of the main path model (Figure 3 in the main text). The priors were default uninformative priors while there were 5,000 warmups, 10,000 iterations within 5 chains. All individual path coefficients with SEs of the model presented in the main text can be found in Table S9. For the sake of clarity, we added the Covid interaction to the path coefficients if necessary (for post-Covid period which was labeled as 1 here) and presented them separately for pre- and post-Covid periods. The differences between the periods, also known as indicators of moderated mediation, are presented next to them in the third part of the table. We use this presentational form throughout the supplement. For the individual values for Covid terms and Covid interactions with other concepts, please see online SI.

The model converged without problems, as can be seen by regular trace and density plots as well as appropriate Geweke and autocorrelations indicators (see online Supplementary Information – online SI: S4). The amount of variance explained in the Outcome, Team Performance, and Referees’ Decisions were high (see R^2^ indicators in Figure 4 in the main text; for the detailed information about proportion of variance explained by fixed, i.e. marginal R^2^, and both fixed and random effects, i.e. conditional R^2^, see online SI). The fit of the model was rather good, as seen by the posterior predictive check, which compares the actual observed values with those that were obtained by the model – see Figure S1.

**Table S9.** Parameters for the main model (main text, Figure 3)

**Figure S1.** Main model posterior checks. Left – Actual distribution (black line) and predicted by the model (blue lines, sample of 100) for the latent variables in the model left. Right – Scatter plot of observed (y-axis) and predicted by model values (y-axis).

**4.1.1 Fixed vs. random effects.** Our study included random effects in the analysis^3^, which enables every team and league to have individual intercepts. Given that the same teams have more similar results than different teams, random effects account for the repeated measure nature and in general controls for random variations in the team performance or general differences between the leagues (e.g., by pulling the estimates for the leagues and teams with fewer data points or with unexpectedly large or small effects (e.g., outliers) towards the estimates from the groups with larger number of observations and the overall mean of the distribution). The fixed effects estimate each team and each league individually without pulling estimates which may or may not result in overfitting individual leagues with fewer data points.

The random effects explain a considerable amount of variance within the results of our models. Figure 4 and the figures here present Bayesian R^2^ (Bayesian coefficient of determination^4^), which also include marginal R^2^, an indication of how much variance random effects explain. Online SI provides detailed information, but suffice to say here that the random part (teams and leagues variations expressed as marginal R^2^) explains between 23% (Referees’ Decisions) and 76% (Dominance) of all the variance (Bayesian R^2^). Intraclass Correlation (ICC), which is an index/correlation of how similar the measures within random units are, also varies between 0.33 (Outcome), 0.46 (Referees), and 0.55 (Dominance) – see Online SI. These results indicate that random effects may add additional value to the fixed effects.

Finally, we provide the main model analysis where hierarchical categories (league and team) are included as fixed effects (see Table S10). The coefficients are remarkably similar, confirming that the overall pattern of results does not depend on the technical solution chosen for the modeling.

We also perform the Hausman test^5^ on the random and fixed models presented in S10. The Hausman test is often taken to indicate whether one should choose models with random or fixed effects^6^. Others argue that the Hausman test cannot be used for such purposes, as it is a test of contextual misspecification such as whether there are other influences (e.g. instrumental variables), or whether within and between effects differ^7,8^. For the sake of completeness, we present the Hausman test in Table S10 (significance indicates that fixed effect should be chosen). There was no difference for the Outcome and Referees’ Decisions parts of the model, but the difference was significant for the Team Performance part. A closer inspection of the random and fixed estimates indicates that almost all coefficients but the one for Rating are remarkably similar. In the random part, the Rating is a strong positive predictor (.05) of Team Performance, whereas there is no association in the fixed model (-.009). Once we remove Rating from the equation, the Hausman test indicates almost a complete equalness of the models (see ALT* in Table S10). The problem seems to be the high association between Team factor and the Rating variable as the two are inevitably almost the same measure (one team will have highly similar rating during the season). When we use the random model without the Team random factor, but with Rating in the equation, it also produces the Hausman test which indicates there are no differences between the random and fixed models (see Online SI).

**Table S10.** Parameters for the main model with random and fixed effects

**4.2. Partial attendance check**

The inclusion of the three leagues (Swiss, Danish, and Russian), which partially allowed a small number of fans into stadiums during the post-Covid period, did not drastically change the pattern of results. It did, however, weaken the strength of the post-Covid period on reducing the HA – see Figure S2. Compared to the main model with no audience, partially allowing audience resulted in a smaller fall in Team Performance from pre- to post-Covid period (from Δ .55 for no audience to Δ .43 for partial audience) and a smaller reversal of referees’ bias (Δ -.49 for no audience to Δ -.38 for partial audience). This led to the fact that the overall HA reduction was statistically reliable only at p < .10 level (Δ -.21 for no audience to Δ -.15 for partial audience). Consequently, it seems that even a handful of supporters considerably improves the Team Performance of the home team and biases referees towards the home team.

**Partial Attendance Model**

**Figure S2.** **Partial attendance model** (includes additional leagues which allowed fans in the post-Covid period). The numbers on the line are path model coefficients. The pre-Covid path coefficients are in blue, the post-Covid coefficients are in red. The statistically reliable coefficients are indicated with *. The difference between the pre- and post-Covid path coefficients is shown above the individual coefficients (*p < .05, **^†^**p < .10 and ns for not significant). Rating (team strength) and Importance (game context) were also included in the model to check for the differences in the schedule strength between pre- and post-Covid periods but were not presented here for the sake of brevity (see online SI).

**4.3. European Championship 2020-21 – further check of partial attendance**

The recently finished European Soccer Championship offers another possibility to check the influence of partially allowing the audience into stadiums. There were 27 matches where one nation was playing in their home stadiums and in front of their fans (usually ¼ of the capacity). The overall advantage of home nations over visiting nations was substantial, even with more than half-empty stadiums. On average, home nations scored 0.44 more goals per match. This is in line with the advantage the home teams had in the qualifications towards the European Championship when they played in the home stadiums full of fans with no restrictions – home teams scored on average 0.44 goals in the qualifications. The advantage is consistent with the advantage the soccer World Cup hosts enjoy when playing in front of full stadiums in the World Cup^9^. The HA is also larger than the HA in the European club competitions in the pre-Covid period as we have shown (Figure 3 in the main text).

There was no association between the home-away goal difference with absolute or relative number of the fans present. Yet, once we controlled for team strength (using 538’s spi rating for national teams), the partial correlation was moderate, *r*_p_ = .28. This indicates that especially weaker home teams profited from the partial presence of the fans.

We also modelled the 27 games using our main model for individual games (see Figure S3) with three exceptions: 1) there was no Covid moderating factor, 2) we also excluded red cards from the Referees’ Decisions as there was only one red card, and 3) we used only Team Rating as control as one could assume that all games were of similar importance for all teams. While the number of games is sufficient for a crude check only, the magnitude of the path coefficients is remarkably similar to those found in the pre-Covid model with fans (see Figure S11). The home teams were performing better, which resulted in better results, and they were less often on the receiving end of official warnings. Most of the coefficients were statistically reliable only at *p* < .10 level, which is not surprising given the small number of observations involved. Please note that according to some prominent figures in the Bayesian movement, the significance level in Bayesian analysis should be set at p > .89 and not p < .95^10^.

Altogether, the Euro 21 results indicate that even a fraction of fans can spur the home team to better performance. Future research should consider that games with partial attendance are most likely different than ghost games.

**EURO 2021 Model**

**Figure S3.** **Euro 2021 model** (partial attendance check. The numbers on the line are path model coefficients. The statistically reliable coefficients are indicated with * and **^†^** (*p < .05, **^†^**p < .10 and ns for not significant). Rating (team strength) was also included in the as a control but were not presented here for the sake of brevity (see online SI). Red cards were not part of the Referees’ Decision as there was only one red card.

**4.4. Robustness check (jackknife resampling)**

The robustness check was done using a jackknife procedure of re-run the same model by leaving out a single league every time. Table S11 demonstrates that the average coefficients on the 10 subsamples are essentially the same as the coefficients in the main model (for the sake of brevity, we left out the control variable coefficients, see online SI). While there were some minor discrepancies (see the minimum and maximum values for individual coefficients in Table S11) the main findings were relatively robust.

For example, the indirect effect of Venue through Team Performance on HA (#14 in Table S11) is statistically reliable in all 10 subsamples in both pre- and post-Covid periods. It’s reduction going from pre- to post-Covid period was also statistically reliable in all subsamples. The total reduction of the HA from pre- to post-Covid period (#16 in Table S11) was found in 9 out of 10 subsamples (even in that instance, the difference was reliable at the .89 level, that is p < .10, a significance level often taken in Bayesian statistics^10^). The reversal of the referees’ bias towards the home team (#19 in Table S11) was also found in all subsamples.

**Table S11.** Average, minimum and maximum parameters for the main model – jackknife procedure of leaving one league out (statistically reliable coefficients are in bold).

**4.5. Main model – Video Assistant Referee (VAR) effect**

The introduction of VAR has arguably made referees’ decisions more objective. Here we checked whether there was a difference between leagues that feature VAR and those that do not regarding referees’ decisions. We added the VAR binary predictor of Referees’ Decision to the main model (see Section 4.1). Figure S4 demonstrates that the overall results are virtually identical to the main model without VAR (Figure 4 in the main text). The VAR variable had a huge positive effect on referees as they reversed the otherwise positive preference of the home team. The estimates were however highly unreliable, resulting in statistically unreliable effects despite their relative magnitude.

One reason for the high unreliability of VAR estimates is that only two leagues did not employ VAR in our sample. Given the magnitude of the VAR effect, research using other non-European leagues, which tend to lack VAR, should include the information explicitly in their models.

**VAR Model**

**Figure S4.** **VAR model** (**Video Assistant Referee**). The numbers on the line are path model coefficients. The pre-Covid path coefficients are in blue, the post-Covid coefficients are in red. The statistically reliable coefficients are indicated with *. The difference between the pre- and post-Covid path coefficients is indicated above the individual coefficients (*p < .05, **^†^**p < .10 and ns for not significant). Rating (team strength) and Importance (game context) were also included in the model to check for the differences in the schedule strength between pre- and post-Covid periods but were not presented here for the sake of brevity (see online SI).

**4.6. Main model – using ratio Fouls/Yellow cards as an indicator of referees’ decisions**

Another way to measure referees’ bias is to use the ratio of numbers of yellow cards a team receives given the number of fouls. A higher ratio would indicate a more stringent application of rules by the referees. In the model presented in Figure S5, we use the standardized ratio of yellow cards and fouls (number yellows / number fouls) instead of the latent referees’ decision variables (based on the variables of fouls, yellow and red cards). The model is consistent with the original model (Figure 4 in the main text) but there are a couple of differences. The reduction in both HA and referees’ bias is also statistically reliable in the new model, but the indirect influence of referees on the outcome and the indirect influence of team performance on the referees did not quite reach statistical significance. The most drastic change is that in the new model the predictors (Venue and Team Performance) of the referees’ bias variable explained almost three times less variance than in the original models (R^2^ = .22 vs. R^2^ = .57). Based on these results, one could argue that a latent variable made of fouls and cards (yellow/red) is more sensitive when it comes to capturing the referees’ bias than the simple ratio of yellow cards and fouls. This might not be surprising given that the latent construct features three individual indicators of referees’ bias. Consequently, the ration yellows/fouls may better capture bias than any of the single referees’ bias indicators, but future research may want to use a latent construct based on all available indicators.

**Yellow/Fouls Ratio Model**

**Figure S5.** **Yellow/Fouls Ratio model** (**Referees’ Decision variable is a ratio of yellow cards received on the number of fouls**). The numbers on the line are path model coefficients. The pre-Covid path coefficients are in blue, the post-Covid coefficients are in red. The statistically reliable coefficients are indicated with *. The difference between the pre- and post-Covid path coefficients is indicated above the individual coefficients (*p < .05, **^†^**p < .10 and ns for not significant). Rating (team strength) and Importance (game context) were also included in the model to check for the differences in the schedule strength between pre- and post-Covid periods but were not presented here for the sake of brevity (see online SI).

**4.7. Main model – alternative pathway Referees’ Decision 🡪 Team Performance**

In the original model we assume that Team Performance affects Referees’ Decisions. In other words, more dominant offensive teams would force less dominant defensive teams to revert to fouling as means of stopping attacks. Given that we use corners and shots as indicators of Team Performance, one could argue that Referees’ Decisions may actually directly lead to shots. An obvious example would be awarding the penalty kick which would inevitably lead to a shot.

A practical consequence of this reversal of the relation between Referees and Team Performance constructs is that the total effect of home venue on Referees’ Decisions may be smaller given that the indirect route through Team Performance is not present anymore. Figure S6 provides the summary of such a model where Referees’ Decisions influence Team Performance (and not vice versa as the model presented in the main text Figure 4). The estimates are virtually identical, and the home court still influences Referees’ Decisions to the same extent as in the (see Venue total in the lower left box in Figure S6). There is no Team Performance to Referees’ Decision path, but its exclusion strengthens the direct path from Venue to Referees’ Decisions (from -.30 pre-Covid in the main model, see Figure 4 main text or Table S9, to -.43 in the same period in the new revised model, see Figure S6 below). Arguably more importantly, the overall effect of home venue on HA (and its reduction from pre- to post-Covid period) is virtually unchanged (see Venue Total in the upper left box in Figure S6 and Figure 4 in the main text).

Theoretically, fouls are most likely a product of team performance in the first place. For example, the possession indicators in the model with additional team performance measures (Figure S16) would be good candidates for capturing team’s dominance, which would precede referees’ decisions. As a matter of fact, we provide such a model in Section 9 (Figure S16). The Possession influences Referees’ Decisions and Attacking tendencies (which include shots and corners), but the Attacking tendencies (shots and corners) do not influence Referees’ Decisions. It can be seen (Figure S16) that the Possession is indeed a better predictor of Referees’ Decisions as its path coefficients are close to -.30 for both pre- and post-Covid periods, compared to around -.10 for the Team Performance in the main model.

The model with possession and attacking/defending indicators may be a more realistic depiction of soccer team performance but is arguably unnecessarily complex. For example, its fit is worse than the fit of the main model, which is not surprising given that the main model features fewer concepts. Pragmatically, one would arguably make a Team Performance latent construct using both possession indicators and shots + corners. We could not do it here as only major leagues had additional statistics freely available.

**Ref −−> TeamPer Model** ****

**Figure S6.** **Referees to Team Performance Model** (**instead of Team Performance to Referees main model**). The numbers on the line are path model coefficients. The pre-Covid path coefficients are in blue, the post-Covid coefficients are in red. The statistically reliable coefficients are indicated with *. The difference between the pre- and post-Covid path coefficients is indicated above the individual coefficients (*p < .05, **^†^**p < .10 and ns for not significant). Rating (team strength) and Importance (game context) were also included in the model to check for the differences in the schedule strength between pre- and post-Covid periods but were not presented here for the sake of brevity (see online SI).

**4.8. Points, Goals, and expected Goals (xG) models**

The Outcome latent variable was composed of two variables, Goals and Points. We also ran separate models for each of these variables instead of the Outcome latent variables. Figure S7 (Points) and Figure S8 (Goals) show that the models using individual predictors of the outcome produce the same pattern of results we already find in the main model. The amount of variance explained was as high as in the main model with the latent variable Outcome (e.g., R^2^ = .36 for Points and R^2^ =.62 for Goals – see Figure S7 and S8). The fit was rather good as seen in Figure S9. The other Bayesian indices showed that the model converged without problems (see online SI).

**Points as Outcome Model**

**Figure S7.** **Points model** (points instead of latent variable Outcome). The numbers on the line are path model coefficients. The pre-Covid path coefficients are in blue, the post-Covid coefficients are in red. The statistically significant coefficients are indicated with *. The difference between the pre- and post-Covid path coefficients is indicated above the individual coefficients (* for significant and ns for not significant). Rating (team strength) and Importance (game context) were also included in the model to check for the differences in the schedule strength between pre- and post-Covid periods but were not presented here for the sake of brevity (see online SI).

**Goals as Outcome Model**

**Figure S8.** **Goals model** (goals instead of latent variable Outcome).

**Figure S9**. Main model posterior checks for goals and points models. Left – Actual distribution (black line) and predicted by the model (blue lines, sample of 100) for the latent variables in the model left. Right – Scatter plot of observed (y-axis) and predicted by model values (y-axis).

We also provide the model on expected goals (xG) instead of goals. Expected goals are calculated based on several variables (distance of shot taken, positions of defenders and goalkeepers) and represent a measure that is less product of luck. As Figure S10 below shows, there is no real difference between the model using the actual goals (Figure S8) and the model using expected goals. If anything, the model with expected goals seems to pronounce the HA reduction as the reduction in the Outcome was larger (from Δ .15 for xG to Δ .10 for the actual goals) and the reversal in referees’ behavior even bigger (from Δ -.67 for xG to Δ -.58 for goals). It is possible that these minor deviations are the consequences of different samples as only the major leagues had available data on expected goals.

**Expected Goals (xG) as Outcome Model**

**Figure S10.** **Expected goals model** (xG instead of latent variable Outcome). The numbers on the line are path model coefficients. The pre-Covid path coefficients are in blue, the post-Covid coefficients are in red. The statistically significant coefficients are indicated with *. The difference between the pre- and post-Covid path coefficients is indicated above the individual coefficients (* for significant and ns for not significant). Rating (team strength) and Importance (game context) were also included in the model to check for the differences in the schedule strength between pre- and post-Covid periods but were not presented here for the sake of brevity (see online SI).

**4.9. Individual games model**

Our main model aggregates individual games across individual teams depending on the Venue and Covid factors. However, it is possible to use individual games as the smallest unit of analysis instead of the averages across the individual teams. Figure S11 demonstrates that the results pattern remains the same when we use the individual games instead of their aggregated means across individual teams. For detailed information about the coefficients, please see online SI.

The model with individual games explained less variance than the main model with averages (from R^2^ = .15 to .21). The explained variance of the variables points out the erratic nature of the individual matches in low scoring games where a single (random) event may change the course of the game (compared to the main aggregated models which uses dozens of individual matches).

The model converged without any problems, but the Outcome variable was not particularly well predicted by the model (see Online SI). This was because the points and goals of an individual game did not follow a normal distribution (transformation and standardization of the variables did not improve the fit). However, the fit improved immensely once these two variables were modelled using non-linear functions (see Online SI).

**Individual Games Model** ****

**Figure S11.** **Individual games model** (with individual games instead of averages across the teams as in the main model). The numbers on the line are path model coefficients. The pre-Covid path coefficients are in blue, the post-Covid coefficients are in red. The statistically significant coefficients are indicated with *. The difference between the pre- and post-Covid path coefficients is indicated above the individual coefficients (* for significant and ns for not significant). Rating (team strength) and Importance (game context) were also included in the model to check for the differences in the schedule strength between pre- and post-Covid periods but were not presented here for the sake of brevity (see online SI).

**4.10. Individual games model + individual referees**

One of the factors that may play a role in the HA, in particular in the Referees’ Decisions, are the individual differences between referees. Some studies ^11–13^ show that there is a great variety in how much individual referees are susceptible to the crowd influence. Therefore, it makes sense to include individual referees as a factor in our model as an additional check. We include the referee names in the model with individual games as it is difficult to include it in the main model where several games have been aggregated. This way, each game is refereed by a single referee which is impossible to achieve in other models. The first model uses referee names as a random factor and produces virtually identical results as when the factor has not been used in Section 4.9 (see Online SI). In the second model we use referee name as a fixed factor. The results of the model with no referee names (from Section 4.9) and the model with referee names as fixed effect is presented in Table S12. The addition of the referee names as the fixed effects hardly changes any estimates with the differences between the estimates of the two models being on the 3^rd^ or 4^th^ decimal place.

**Table S12.** Parameters for the individual games model without individual referees as a factor and with individual referees modeled as a fixed effect.

1. **Rating (team strength) and Importance – Schedule Strength Check**

To control for possible differences in the pre- and post-Covid schedule, we used the strength of individual teams and the game importance for individual teams. If importance and strength of away teams was significantly higher than that of home teams in the post-Covid period, this would undermine the importance of audience. FiveThirtyEight’s team strength rating (SPI) includes teams’ previous results, market values of players, and it is updated after each game based not only on the actual results and goals scored, but also on other indicators to account for randomness of a low scoring game such as soccer (e.g., adjusted goals, shot-based expected goals and non-shot expected goals). FiveThirtyEight’s importance measures the impact of the match results on the team's outlook on the season. The importance depends on the team, as different teams play for the championship, qualification for international UEFA competitions, or not getting relegated. Similarly, the importance measure takes the situation in the individual league into account as the probabilities of achieving a team's goal are calculated depending on the outcome of the game. The difference between the probabilities is then expressed as a standardized variable. Both rating and importance measures range from 0 to 100 and are comparable not only within a single league, but also across the leagues.

The simplest schedule check would be a Venue x Covid ANOVA on the Rating and Importance variables (see also Table S1 for averages of the individual cells). If there is a significant interaction between Venue and Covid, one could infer the differences in the schedule. This is neither the case for Rating (F(1,8382) = .03, p = .86) nor for Importance (F(1, 8382) = .01, p = .93).

A more sophisticated check would be the interaction of Rating and Importance variables with the variables Covid within the main Bayesian model. Again, if there are any discrepancies between pre- and post-Covid schedule for home and away teams, this interaction should capture it. Just like the simple ANOVA above, the interaction with Covid was not significant either for Rating or Importance (see – Section 4.1 – Main Model, Table S9).

1. **Alternative Analysis – Path Analysis**

The same path models in the main text (see also Section 4 here) can be run in the frequentist framework. Here we used piecewiseSEM R package^14^ for conditional path analysis ^15^. Given that the model is saturated (i.e., all possible relations have been specified), the fit indices (e.g. CFI, TLI, or RMSEA) were not available. The frequentist path model however, explained moderate amounts or variance as the main Bayesian model: R^2^ conditional was 0.69, 0.60, and 0.54 for Outcome, Team Performance, and Referees’ Decisions, respectively (see online SI).

As Table S13 shows, the results are practically the same as in the main model. Again, the largest effect is the interaction of Covid and Venue on Team Performance; the Covid x Venue interaction for Referees’ Decisions is significant. The disadvantage of this approach is that it is not straightforward to calculate indirect effects and the differences between pre- and post-Covid periods. We do, however, provide in online SI an alternative way for calculating these effects in the frequentist framework. The online SI also provides the analyses presented in Section 4 (e.g., individual games model, model for points and goals separately) in the frequentist framework.

**Table S13.** Frequentist path analysis parameters for the main model

1. **Alternative Analysis – SEM**

The main analysis in Section 4 (and its frequentist counterpart in Section 6) used composites of the variables (e.g., Outcome was composed of Goals and Points). One could also directly use these variables to produce latent variables in the Structural Equation Modeling (SEM) framework ^16^. The analyses were done in lavaan R package^17^ using Team variable as the cluster index (repeated measures, or smallest units) and Covid as grouping variable (pre and post – see SOM for more details on the model) on individual games. Besides the well-known relations, we also added bilateral relations between individual variables such as Goals and Shots on target (see Table 10) to improve model fit.

The SEM model was able to converge without problems and had adequate fits CFI = .97 and TLI = .94; RMSEA was .046 with 90CI of .043 and .050, p > .05; SRMR was .029. The predictors explained between 56 and 73% of Output (depending on the Covid period), 13 and 21% of Referees’ Decisions, and 13 and 39% of Team Performance. While the amount of explained variance was smaller than in the path models, the conducted SEM produces essentially the same results as the main model (see Table S14).

The online SI also reports the results of a SEM model with individual games, which also confirms the pattern of results we find in the models with aggregated data.

**Table S14.** Frequentist SEM main model

1. **Alternative Approach – Home-Away difference model**

Our main model (Section 4 and the main text) used the aggregated data across teams and their individual games as the lowest level of analysis. Another way of analyzing the data is to have the difference between home and away team directly in the model. The other variables in the model are also using differences between home and away teams instead of their raw values. For example, Team Performance is now an indicator how big the dominance of the home team compared to that of the away team was: the differences between corners for home and away team, as well as for the shots, and shots on target are now providing the input variables for the latent factor. The same procedure is done for the Referees’ Decision and Outcome. The model is the same as in the main text, except that now there is the Covid factor instead of the Venue factor (Figure S12).

As seen in Figure S12, the results are essentially the same as in the main model. The detailed results can be found in the online SI. The model had a good fit as seen by posterior checks for the main concepts in Figure S13.

**Differences (Home – Away) Model**

**Figure S12.** **Home-Away differences model** (with hoe-away differences instead of averages across the teams as in the main model). The numbers on the line are path model coefficients. The statistically significant coefficients are indicated with *. The difference between the pre- and post-Covid path coefficients is indicated above the individual coefficients (* for significant and ns for not significant). Rating (team strength) and Importance (game context) were also included in the model to check for the differences in the schedule strength between pre- and post-Covid periods but were not presented here for the sake of brevity (see online SI).

**Fig. S13.** Main model posterior checks for the alternative differences (home-away) model. Left – Actual distribution (black line) and predicted by the model (blue lines, sample of 100) for the latent variables in the model left. Right – Scatter plot of observed (y-axis) and predicted by model values (y-axis).

1. **Alternative Models with Additional Team Performance Indicators**

In Section 3, we have seen that the team performance indicators form other factors besides attack/dominance. These factors, most notably possession and defense/set-pieces were included here in models. Theoretically, it makes sense to assume that attacking and defending tendencies depend on how much a team has the ball in their possession. This was also the only model with three latent factors of team performance factors that fitted the data well. We therefore created a model where the possession factor preceds both attack and defense factors (see Figure S14). We also assumed that only possession influences Referees’ Decisions, not attack or defense (the model where all three influence Referees’ Decisions was not fitting better).

Figure S14 shows that we have the same pattern of results as when we use only attack/dominance as the Team Performance factor in the main analysis. The model converged well (see online SI), but it did not fit significantly better than the main model with only attack/dominance as the Team Performance indicator in the main analysis (see online SI). Consequently, we have chosen the simpler model for the main presentation.

**Additional Team Performance Indicators Model**

**Figure S14.** **Additional team parameters model.** The team indicators were captured by three factors: Possession, Defense (set pieces), and Attack (Team Performance in the main model). The numbers on the line are path model coefficients. The pre-Covid path coefficients are in black, the post-Covid coefficients are in red. The statistically significant coefficients are indicated with *. The difference between the pre- and post-Covid path coefficients is indicated above the individual coefficients (* for significant and ns for not significant). Rating (team strength) and Importance (game context) were also included in the model to check for the differences in the schedule strength between pre- and post-Covid periods but were not presented here for the sake of brevity (see online SI). Similarly, the Venue 🡪 Defense relations was omitted here to avoid clutter (see online SI).

1. **Distance (Fatigue), Attendance, and Stadium Analysis**

**10.1 Descriptive Statistics**. Travel fatigue is often taken as another factor contributing to the HA as away teams need to travel to the venue. Correlations between the distance traveled of the away team and the difference between home and away teams in the latent factors of Outcome and Team Performance virtually shows no associations (Table S15). How much the away team traveled, was not associated with how worse they performed and fared compared to the home team.

**Table S15**. Intercorrelations between distance, audience, and stadium variables on the one hand, and the home-away difference in Outcome, Referees’ Decisions, and Team Performance on the other.

The variable audience could be refined if one takes into account their absolute (attendance) and relative numbers (ratio between attendance and capacity, i.e. attendance density) as well as the characteristics of the stadium such as presence of the running track (i.e. proximity of the audience), size of the pitch, and kind of grass (natural vs. artificial). The absolute audience size (Attendance) was positively correlated with the difference between home and away teams when it comes to Outcome and Team Performance. It was negatively associated with the differences in Referees’ Decision – the more fans were in the stadium, the better the home team fared compared to the away team, the better it performed, and it received fewer official warnings. These associations were rather small, but have reached the significance level due to the number of games (over 4,000).

However, these associations are deceiving as stronger teams in general have bigger stadiums and more attending fans. This can be seen in the correlation between Attendance and Rating (*r* = .18), which indicates that the bigger the difference in the strengths of home and away teams, the bigger the attendance (the fans also went to the stadium when the game was of bigger importance for the home team – see Attendance correlation with Importance). Similarly, when we consider the relative number of fans, that is attendance density, the associations with the Outcome and Team Performance were considerably weaker (and in the first case not significant). Stronger teams also have larger stadiums, which are mostly full, which in turn weakens the association of the relative attendance with the outcome.

The same pattern can be seen for the presence of the Running Track, which indicates whether the stadium has the audience closer or further from the players. The home teams playing in the stadiums with the Running Track are less successful, which may not come as a surprise as those teams are also weaker teams (see the relations between Running Track and Rating) and do not have their own, soccer dedicated stadiums.

**10.2 Distance and attendance in models**. Arguably a more appropriate way of checking for the influence of these additional variables would be their inclusion in the model. This is difficult to achieve in the main model which features the Venue factor. Venue is highly correlated with both distance and attendance, which makes these variables redundant in the model. The distance can be integrated in the model with home-away differences (see Section 8) and it does not impact neither the Outcome directly, nor it does impact the Team Performance – see Table S16.

**Table S16.** (Bayesian) Distance model

Attendance, however, cannot be incorporated into this model as it is directly related to the Covid period (i.e. no fans in the post-Covid period). We did include the Attendance in the model featuring only the set of pre-Covid games in the model with differences between home and away teams (see Section 8). That way one can get a better insight into how much absolute and relative attendance influenced the differences between home and away teams.

Table S17 shows that the absolute number of fans influences (significantly) positively the direct outcome but is not a significant predictor of team performance nor does it bias referees’ decisions. The relative attendance (fans/stadium capacity), however, is not only irrelevant for team performance and referees’ decisions, but it also has no significant effect directly on the outcome – Table S18.

**Table S17.** (Bayesian) Attendance model (absolute)

**Table S18.** (Bayesian) Attendance model (relative – density)

**SI References**

1. Park, Y.-S., Choi, M.-S., Bang, S.-Y. & Park, J.-K. Analysis of shots on target and goals scored in soccer matches: implications for coaching and training goalkeepers. *South Afr. J. Res. Sport Phys. Educ. Recreat.* **38**, 123–137 (2016).

2. Bürkner, P.-C. brms: An R package for Bayesian multilevel models using Stan. *J. Stat. Softw.* **80**, 1–28 (2017).

3. Gelman, A. & Hill, J. *Data analysis using regression and multilevel/hierarchical models*. (Cambridge university press, 2006).

4. Gelman, A., Goodrich, B., Gabry, J. & Vehtari, A. R-squared for Bayesian regression models. *Am. Stat.* **73**, 307–309 (2019).

5. Hausman, J. A. Specification tests in econometrics. *Econom. J. Econom. Soc.* 1251–1271 (1978).

6. Wooldridge, J. M. *Econometric analysis of cross section and panel data*. (MIT press, 2010).

7. Bell, A., Fairbrother, M. & Jones, K. Fixed and random effects models: making an informed choice. *Qual. Quant.* **53**, 1051–1074 (2019).

8. Fielding, A. The role of the Hausman test and whether higher level effects should be treated as random or fixed. *Multilevel Model. Newsl.* **16**, 3–9 (2004).

9. Torgler, B. ‘Historical Excellence’ in Football World Cup Tournaments: Empirical Evidence with Data From 1930 to 2002. *CREMA Work. Pap.* (2004).

10. Kruschke, J. K. *Doing Bayesian data analysis: a tutorial with R and BUGS*. (Academic Press, 2011).

11. Boyko, R. H., Boyko, A. R. & Boyko, M. G. Referee bias contributes to home advantage in English Premiership football. *J. Sports Sci.* **25**, 1185–1194 (2007).

12. Goller, D. & Krumer, A. Let’s meet as usual: Do games played on non-frequent days differ? Evidence from top European soccer leagues. *Eur. J. Oper. Res.* **286**, 740–754 (2020).

13. Page, K. & Page, L. Alone against the crowd: Individual differences in referees’ ability to cope under pressure. *J. Econ. Psychol.* **31**, 192–199 (2010).

14. Lefcheck, J. S. piecewiseSEM: Piecewise structural equation modelling in r for ecology, evolution, and systematics. *Methods Ecol. Evol.* **7**, 573–579 (2016).

15. Shipley, B. *Cause and correlation in biology: a user’s guide to path analysis, structural equations and causal inference with R*. (Cambridge University Press, 2016).

16. Kline, R. B. *Principles and practice of structural equation modeling*. (Guilford publications, 2015).

17. Rosseel, Y. Lavaan: An R package for structural equation modeling and more. Version 0.5–12 (BETA). *J. Stat. Softw.* **48**, 1–36 (2012).
